# Chronic scrotal heat stress causes testicular interstitial inflammation and fibrosis: An experimental study in mice

**DOI:** 10.18502/ijrm.v20i7.11559

**Published:** 2022-08-08

**Authors:** Tung Nguyen-Thanh, Phuoc Dang-Van, Phuc Dang-Ngoc, Won Kim, Tam Le-Minh, Quoc-Huy Nguyen-Vu

**Affiliations:** ^1^Faculty of Basic Science, Hue University of Medicine and Pharmacy, Hue University, Hue, Vietnam.; ^2^Institute of Biomedicine, Hue University of Medicine and Pharmacy, Hue University, Hue, Vietnam.; ^3^Forensic Medicine Center in Thua Thien Hue Province, Hue, Vietnam.; ^4^Faculty of Medicine, Dong A University, Da Nang, Vietnam.; ^5^Department of Internal Medicine, Jeonbuk National University Medical School, Jeonju, Republic of Korea.; ^6^Department of Obstetrics and Gynecology, Hue University of Medicine and Pharmacy, Hue University, Hue, Vietnam.

**Keywords:** Heat stress, Testicular, Inflammation, Fibrosis.

## Abstract

**Background:**

Chronic heat stress is a risk factor that adversely affects the reproduction system. Inflammation and fibrosis are 2 important response processes to damaged tissues.

**Objective:**

This study investigates the association of chronic scrotal heat stress with testicular interstitial inflammation and fibrosis in mice.

**Materials and Methods:**

For all experiments, 8-10 wk old male Swiss mice (*Mus musculus*) (20-23 gr) were divided into 3 groups (n = 10/each). The heat-stress groups were submerged in a water bath at 37 C and 40 C, while the control group was treated at 25 C. The testicular tissues underwent hematoxylin and eosin staining, picro sirius red staining, and immunohistochemistry for intercellular adhesion molecule-1, fibroblast-specific protein 1, F4/80, collagen I, and Ki-67 staining to determine the testicular interstitial inflammation and fibrosis.

**Results:**

Chronic scrotal heat stress impairs spermatogenesis and reverses testicular histological structure. In this study, heat stress significantly induced increased interstitial cell proliferation and upregulation of intercellular adhesion molecule-1 expression in the interstitial testicular tissue. In the interstitial testicular tissue, the number of F4/80-positive macrophages and the number of fibroblast-specific protein 1-positive fibroblasts were significantly increased in the heat-exposed groups compared to those in the control group. The heat exposed groups had substantially increased extracellular matrix collagen accumulation in their testicular interstitial tissues.

**Conclusion:**

Heat stress adversely affects the testicular structure and spermatogenesis, causes inflammation, and leads to testicular interstitial fibrosis.

## 1. Introduction

The scrotum temperatures of mammal animals are often lower than body temperature to accommodate normal spermatogenesis (1). Heat stress is a harmful factor for many biological systems in the body, such as the circulatory system, integumentary system, respiratory system, and male reproductive activity (2). An increase in scrotal temperatures in male mammal animals and men results in reduced sperm parameters, such as decreased sperm motility, and increases in the percentage of sperm with abnormal morphology (3).

There are many factors that affect spermatogenesis. Heat stress causes cell and molecular changes, affecting gene expression that disrupts sperm production, resulting in reduced reproductive health (4). It has been determined that spermatogenic cells are sensitive to high temperatures (2). After testicular heat stress, many cells undergo extrinsic and intrinsic apoptosis signal pathways (5). Spermatogenic cells are affected by heat stress principally by primary spermatocytes and early spermatids (6). In a previous study, the heat-exposed mice showed degenerative changes with spermatic arrest in most of the seminiferous tubules (7). Heat stress has also been shown to increase sperm DNA fragmentation (8). The mechanism of sperm DNA damage may involve various types of oxidative reactions, DNA repair errors in the late stages of spermatogenesis, and functional abnormalities that reduce the protective ability of Sertoli cells (9).

Heat stress adversely affects human and animal health, including through an increase in the inflammatory process in the body, largely in mammals (10). Several studies have shown that heat stress increases inflammatory responses and the release of inflammatory mediators such as cytokines in the kidney, muscle, and intestine (11, 12). Heat stress has been shown to systemically increase inflammation signals through the pathways of toll-like receptor 4, activator protein 1, and nuclear factor kappa-light-chain-enhancer of activated B (13, 14).

Several studies have shown that heat stress affects the induction of fibrosis in various tissues including muscle, liver, and lung (15-17). Heat stress increases the excessive accumulation of extracellular components, in which collagen plays an important role, and this process often occurs after trauma; inflammation leads to increased fibrosis, resulting in reduced function and causing chronic diseases in many tissues and organs (18). In addition, Dos Santos Hamilton and colleagues showed that there was a mild multifocal interstitial fibrosis in ram testicular tissue exposed to heat stress (8).

Currently, studies on the effects of heat stress on testicular inflammation and fibrosis are limited. This study has investigated the histopathological changes related to inflammation and fibrosis in response to testicular chronic heat stress.

## 2. Materials and Methods

### Animals

This experimental study was performed on a mice model. Animals were kept in an animal facility at a controlled temperature (25 ± 1 C) and illuminated (12 hr light/dark cycle) with free access to food and water. Thirty 8-10 wk old male Swiss mice (*Mus musculus*) (20-23 gr) were divided into 3 groups (n = 10/each). The heat-stress groups were submerged in a water bath at 37 C (H37 C group) and 40 C (H40 C group), while the control group was treated at 25 C.

### Induction of chronic heat stress

The lower body of the mice including the scrotum was soaked for 10 min in a temperature-controlled water heated bath twice a day, 10 min apart, for 5 consecutive wk, and 6 days a wk. The mice were checked for wounds or redness after being soaked and dried, then returned to the cages of each group. The mice were raised under the same environmental conditions, given free food and enough water, and monitored for general health.

### Hematoxylin and eosin staining

The animal was sacrificed after 5 wk of heat stress. The testis' tissues were harvested for histological analysis. The testicular specimens were individually immersed into 4% buffered formaldehyde and dehydrated with graded concentrations of ethanol then embedded into paraffin. The paraffin blocks were cut thinly with a thickness of 5 µm and transferred into gelatinized slides. The sections were deparaffinized with xylene and then rehydrated through a descending series of ethanol and water. Slides were stained with hematoxylin and eosin and then observed under a light microscope (19).

### Johnsen scoring system

Testicular and sperm lesions were assessed with a testicular average score according to Johnsen (20). By using a × 40 magnification, 30 testicular tubules for each animal were graded. Each tubular section was given a score from 1-10 according to the presence or absence of spermatogenous cells in the lumen as spermatozoa, spermatids, spermatocytes, spermatogonia, and Sertoli cells. The state of spermatogenesis was assessed as normal or good with a high Johnsenian score, in contrast to poor fertility or dysfunction of the semen function with a low Johnsenian score. A score of 10 means that all types of sperm cells arranged in an order in the lumen of the spermatogenesis indicate normal spermatogenesis, while a score of 1 indicates no epithelial maturation (Table I).

### Picro sirius red staining

To evaluate the collagen accumulation, paraffin-embedded tissue sections were stained with picro sirius red and quantified by image analysis as described previously (21). Briefly, the sections were deparaffinized, hydrated, and then incubated with 0.1% picro sirius red solution (Sigma Chemical Co., St. Louis, MO, USA) for 1 hr. Tissue sections were cleared in xylene and mounted in a resinous medium. The ImageJ software (http://rsb.info.nih.gov/ij) was used to measure 10 randomly chosen non-overlapping fields of the picro sirius red-positive areas.

### Immunohistochemistry staining

Immunohistochemical staining was performed as described previously (21). Briefly, the tissue sections were boiled in citrate buffer (Dako target retrieval solution, S1699, DAKO, Carpenteria, CA) to achieve antigen retrieval for 20 min. The slides were then incubated with peroxidase blocking solution (S2023, DAKO) for 15 min and protein block serum-free (X0909, DAKO) for 30 min. Tissue sections were incubated overnight at 4 C with primary antibodies: type I collagen (1:200; 1310-01; Southern Biotech, Birmingham, AL, USA), intercellular adhesion molecule-1 (ICAM-1) (1:200; sc1511; Santa Cruz Biotechnology, Santa Cruz, CA, USA), F4/80 (1:200; 14-4801-81; eBioscience, San Diego, CA, USA) and fibroblast-specific protein 1 (FSP-1)/S100A4 (1:200; ab41532; Abcam, Cambridge, UK). Second antibodies were incubated for 30 min at room temperature and then treated with horseradish peroxidase-conjugated streptavidin (P0397, DAKO) for 30 min. To visualize the immunocomplexes, the testicular sections were treated with 3-amino-9-ethyl carbazole substrate solution (K3464, DAKO). The F4/80-positive macrophages and FSP1-positive fibroblasts per high-power field were counted at a magnification of × 200. The ImageJ software was used to measure the fibrotic areas of type I collagen and ICAM-1 positive area in 10 randomly chosen non-overlapping fields at a magnification of × 200.

**Table 1:** The Johnsen scoring system for histological classification of testicular seminiferous tubules of the testis (20)


**Score**	**Description**
**10**	Complete spermatogenesis with many spermatozoa. Germinal epithelium is organized in a regular thickness leaving an open lumen
**9**	Many spermatozoa present but the germinal epithelium is disorganized with marked sloughing or obliteration of the lumen
**8**	Only a few spermatozoa present in the section
**7**	No spermatozoa but many spermatids present
**6**	No spermatozoa and only a few spermatids present
**5**	No spermatozoa, no spermatids but several or many spermatocytes present
**4**	Only a few spermatocytes and no spermatids or spermatozoa present
**3**	Spermatogonia are the only germ cells present
**2**	No germ cells but Sertoli cells present
**1**	No cells in the tubular section

### Ethical considerations

The animal experiment protocol was reviewed and approved by the Ethical Committee of Hue University of Medicine and Pharmacy, Hue, Vietnam (Certificate no. H2019/345). Mice were provided by the Pasteur Institute of Nha Trang, Vietnam.

### Statistical analysis

Statistical analysis was performed using Predictive Analytics Software statistic 18 (SPSS Inc., Chicago, IL). The student's *t* parametric test was used to assess statistical significance between the means of 2 independent groups with normal distribution, while data without normal distribution were analyzed using the nonparametric Mann-Whitney U test. All values are presented as mean ± standard deviation. A p-value < 0.05 was considered statistically significant.

## 3. Results

### Heat stress impairs spermatogenesis in the testis

**Figure 1:**
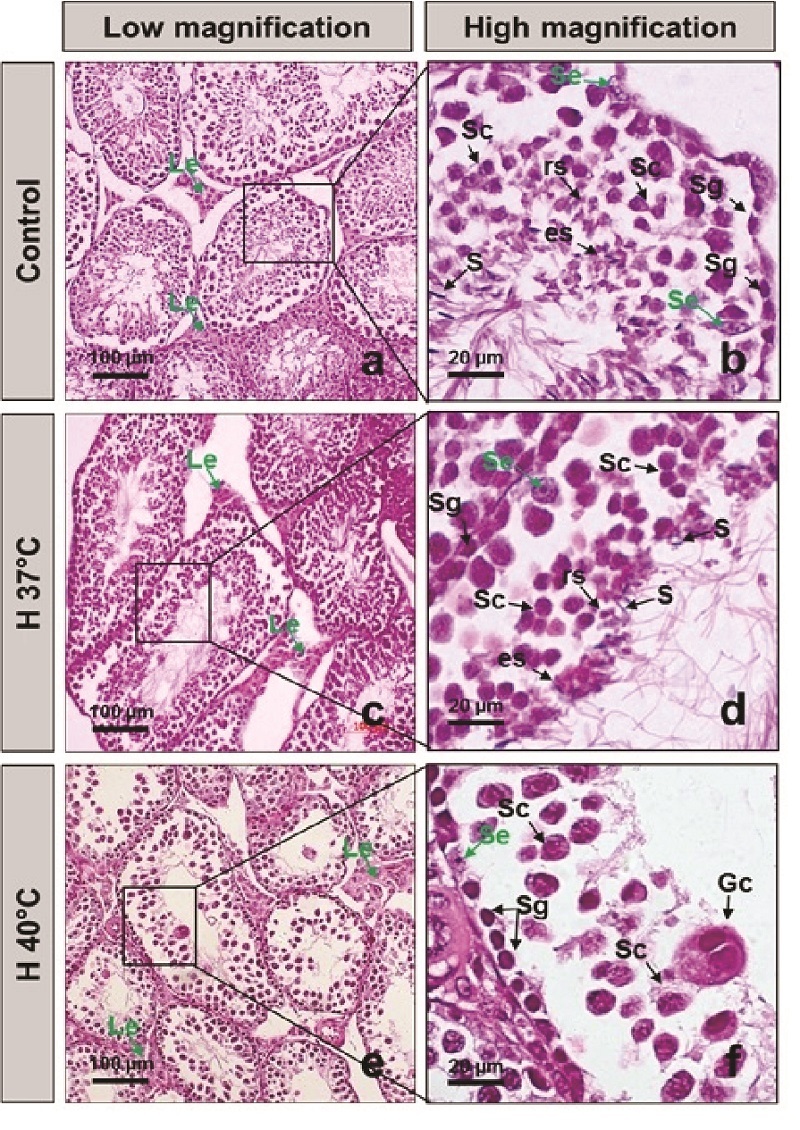
Testicular structure and spermatogenesis in the normal and heat-stress-exposed male mice. a-b: Testicular structure and spermatogenesis in the normal male mice (control). c-d: Testicular structure and spermatogenesis in the 37 C heat-stress-exposed male mice (H37 C). e-f: Testicular structure and spermatogenesis in the 40 C heat-stress-exposed male mice (H37 C). Le: Leydig cells, Se: Sertoli cells, Sg: Spermatogonia, Sc: Spermatocytes, rs: Round spermatids, es: Elongated spermatids, S: Spermatozoa, Gc: Multinucleated giant cells.

Normal mice without heat exposure showed histopathology of normal testicles with a full structure of spermatogenous cells arranged in order into the lumen (Figure 1a). The spermatogenetic cells were located within invaginations of Sertoli cells. The germinal epithelium included different developmental stages of germ cells, namely spermatogonia, primary and secondary spermatocytes, round and elongated spermatids, and spermatozoa (Figure 1b).

**Figure 2:**
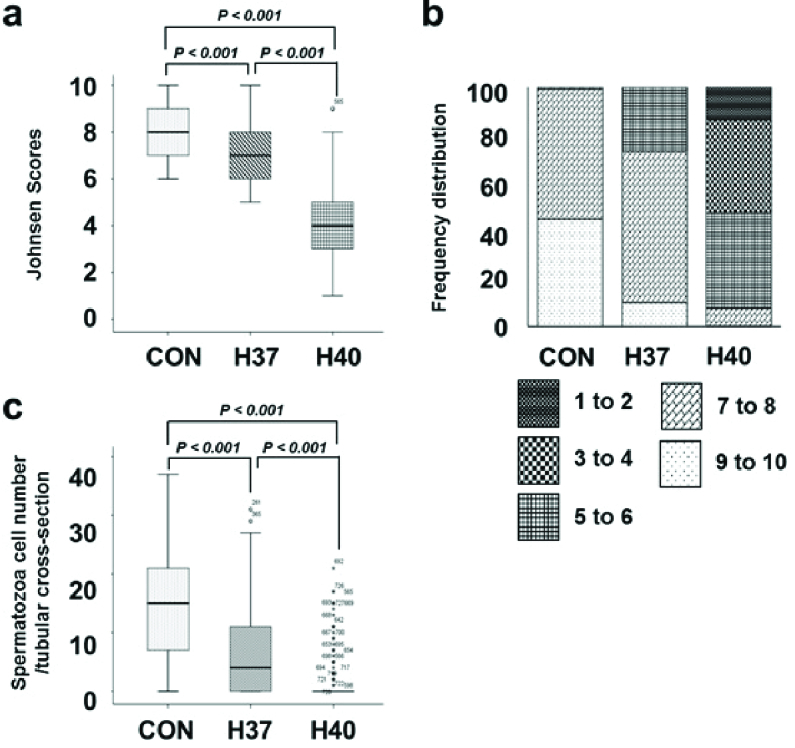
Heat stress induces histopathology and spermatogenesis changes in the testis. a: Johnsen score of seminiferous tubular cross-sections in the normal and heat-stress-exposed male mice. b: Frequency distribution of the Johnsen scores of seminiferous tubular cross-sections in the normal and heat-stress-exposed male mice. c: Spermatozoa number per tubular cross-section in the normal and heat-stress-exposed male mice. CON: Control group, H37: 37 C heat exposure group, 40 C heat exposure group.

Meanwhile, the heat-stress-exposed mice (37 C and 40 C) exhibited degenerated and disorganized features of spermatogenic epithelium and reduced spermatogenic cell numbers (Figures 1c, d, e, and f). In the H40 C group, the complete arrest of spermatogenesis at the spermatocyte stage was the main feature. There were several multinucleated giant cells in seminiferous tubules (Figure 1f). Chronic heat exposure at 37 C and 40 C for 5 wk impaired the histological architecture of the testicular tissue, resulting in a significantly reduced Johnsen score compared to the control group (Figure 2a), decreased ratio of high Johnsen score, and a gradual increase in the percentage of the low score (Figure 2b). Spermatozoa counted per seminiferous tubule was significantly reduced in the heat-stress-exposed group compared to those in the control group (Figure 2c).

### Heat stress induces testicular interstitial cell proliferation and inflammation in the testis

**Figure 3:**
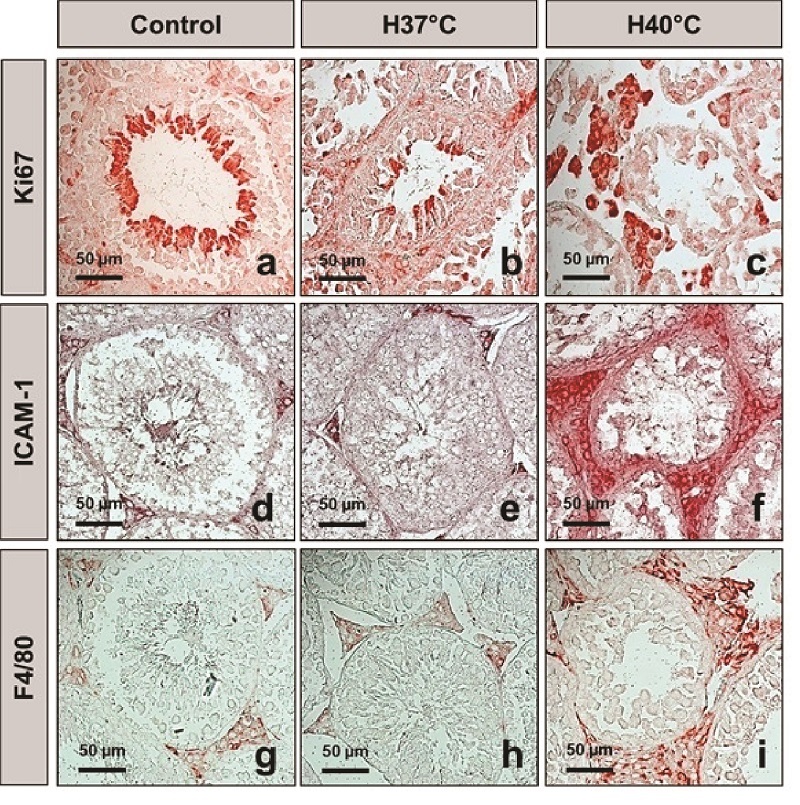
Expression of cell proliferation marker Ki-67, intercellular adhesion molecule-1, and macrophage marker F4/80 in the interstitial tissue of testes after heat stress. a-c: Immunohistochemical staining for Ki-67 in the normal and heat-stress-induced testicular tissue. d-f: Intercellular adhesion molecule-1 expression in the testicular interstitial tissue. g-i: F4/80 macrophage marker detected by immunohistochemical staining. Control: Control group, H37 C: 37 C heat exposure group, H40 C: 40 C heat exposure group, ICAM-1: Intercellular adhesion molecule-1.

**Figure 4:**
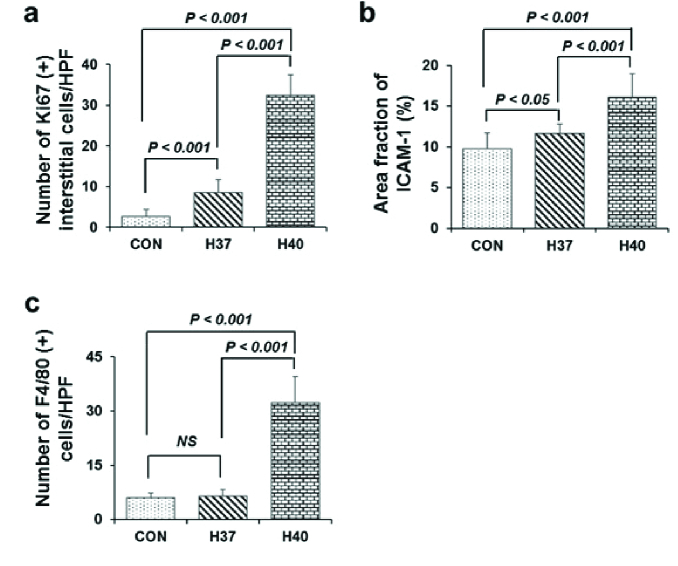
Immunohistochemical analysis of the expression of Ki-67, intercellular adhesion molecule-1, and F4/80 in the heat-stress-induced testicular interstitial tissue. a: The number of Ki-67-positive interstitial cells per high-power field. b: Histogram analysis of the area fraction of intercellular adhesion molecule-1. c: The number of F4/80-positive cells per high-power field. HPF: High-power field, NS: Not significant, CON: Control group, H37: 37 C heat exposure group, H40: 40 C heat exposure group, ICAM-1: Intercellular adhesion molecule-1.

Ki-67 marker was used to determine the cell proliferating in the normal and heat-stress-exposed testes (Figures 3a, b, c). There were many round and elongated spermatids positive with Ki-67 in the intra-seminiferous tubules in the control group. Meanwhile, there were no Ki-67-positive cells inside the seminiferous tubules in the H40 C group. In contrast, heat stress induced increased interstitial cell proliferation. In the H40 C group, many interstitial cells positive with Ki-67 were observed inside testicular interstitial tissue. The heat-stress-exposed groups (H37 C and H40 C) had significantly increased numbers of Ki-67-positive cells in the testicular interstitial tissue compared to the control group (p < 0.001) (Figure 4a).

ICAM-1 plays a key role in the induction and development of inflammation. The expression of ICAM-1 in testicular tissues was investigated at 5 wk after heat treatment. Immunohistochemistry staining of ICAM-1 is shown in figures 3d, e, and f. In the H40 C group, ICAM-1 had a strong expression in the cytoplasm and membrane of the testicular interstitial cells (Figure 3f). The total area fraction of ICAM-1 was quantified as shown in figure 4b. Heat stress induced at 40 C significantly increased the area fraction of ICAM-1 compared to those in the control and 37 C groups (p < 0.001).

Macrophage marker F4/80 expression in testicular interstitial cells is shown in figures 3g, h, and i. Heat stress exposure at 40 C strongly induced F4/80 expression in the cytoplasm and membrane of the testicular interstitial cells (Figure 3i). Heat stress exposure at 40 C significantly increased the number of F4/80-positive macrophages in the testicular interstitial tissues compared to in the control and H37 C groups (p < 0.001). Meanwhile, the number of F4/80-positive macrophages in the testicular interstitial tissues in the H37 C group did not increase compared to those in the control group (Figure 4c).

### Testicular interstitial fibrosis is activated following scrotal heat stress

**Figure 5:**
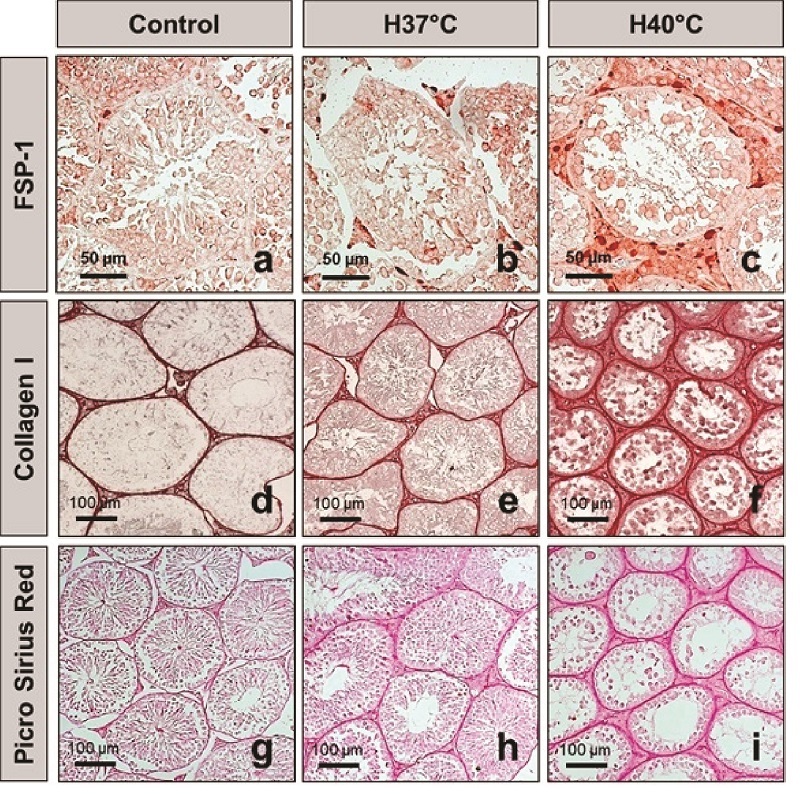
Evidence of fibrosis in testicular interstitial tissue after heat stress exposure. a-c: Immunohistochemical staining for fibroblast-specific protein 1. d-f: Immunohistochemical detection of collagen I deposition following scrotal heat stress. g-i: Collagen network detected by picro sirius red staining. Control: Control group, H37 C: 37 C heat exposure group, H40 C: 40 C heat exposure group, FSP-1: Fibroblast-specific protein 1.

**Figure 6:**
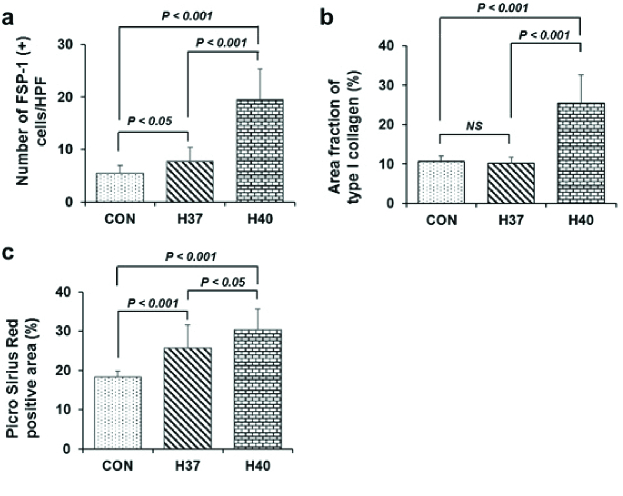
Interstitial fibrosis measurement of heat-stress-induced testicular tissue. a: Histogram analysis of the number of FSP-1-positive cells per high-power field. b: Histogram analysis of area fraction of collagen I. c: Picro sirius red-positive area analysis. HPF: High-power field, NS: Not significant, CON: Control group, H37: 37 C heat exposure group, H40: 40 C heat exposure group, FSP-1: Fibroblast-specific protein 1.

FSP-1 is considered a fibroblast-specific marker expressed in normal and fibrotic tissues. Interstitial FSP-1-positive fibroblasts were detected by immunohistochemistry as shown in figures 5a, b, and c. The number of FSP-1-positive fibroblasts in the testicular interstitial tissue was significantly increased in the H40 C group compared to those in the control and H37 C groups (p < 0.001) (Figure 6a).

Immunohistochemical staining of collagen I deposition following scrotal heat stress is shown in figures 5d, e, and f. Extracellular matrix collagen I fibrils accumulation was strongly detected in the interstitial tissue of testes exposed at 40 C (Figures 5d, e, f). The collagen I area fraction was significantly increased in the H40 C group compared to that of the control group (p < 0.001). Meanwhile, heat stress exposure at 37 C did not increase the area fraction of collagen I compared to that of the control group (Figure 6b).

Histological visualization of collagen I and III fibers by picro sirius red staining is shown in figures 5g, h, and i, and figure 6c. Collagen fiber accumulation in testicular interstitial tissue was significantly induced in the heat-stress-exposed groups (H37 C and H40 C) compared to in the unexposed group (Figure 6c).

## 4. Discussion

This study showed that heat stress exposed degenerated and disorganized features of the spermatogenic epithelium and reduced spermatogenic cell numbers in mice. In the heat-stress-exposed groups, the complete arrest of spermatogenesis at the spermatocyte stage was the main feature. Previous studies have shown that an increase in testicular temperatures in mammals impairs spermatogenesis, damages germ cells, decreases sperm motility, and increases the percentage of sperm with abnormal morphology (3, 22, 23). The effect of heat stress on testicular function and fertility in mice is also shown in Paul and colleagues' research (24). In addition, heat stress increases free radicals, oxidative stress, and increased sperm DNA fracture resulting in apoptosis of spermatogenic cells, and loss of sperm integrity.

Inflammation is the body's beneficial response to the elimination of damaging effects to the body. And inflammation should be actively tackled to avoid leading to chronic inflammation, tissue destruction, and progression to fibrosis (25). The damaged organization releases chemical factors that attract inflammatory white blood cells to the site of the lesion to increase the production of proinflammatory chemokines and cytokines, and these cells also secrete chemical intermediates which are harmful to the surrounding tissue; in addition, chronic inflammation causes fibrosis when severe, repetitive tissue damage or damage is disrupted, resulting in excessive buildup of fibrous connective tissue in tissue damage leading to fibrosis (26). In addition, the failure of inflammatory regulatory mechanisms to address inflammation results in chronic inflammation, persistent tissue damage, and fibrosis (25).

Heat stress increases inflammation in the testis by activating proinflammatory cytokines (interleukin 1 beta and tumor necrosis factor-alpha) and increasing their expression (27). This increases the migration of leukocytes into the reproductive system and increases the production of reactive oxygen species leading to oxidation imbalances, resulting in disorders of spermatogenesis, decreased sperm quality, and infertility (28, 29).

Immunohistochemical staining for ICAM-1 showed the heat-stress-induced inflammatory interstitial cells in the testis. The H40 C group had significantly increased numbers of F4/80-positive macrophages per high-power field in the testicular interstitial tissue compared to the control and 37 C groups. These results are consistent with earlier research; ICAM-1 has been implicated in a variety of inflammatory responses in testicular tissue, tumor cells, and synovial tissues (30, 31).

Ki67 cell proliferation marker staining in figure 2 shows that heat stress disrupted intra-seminiferous tubule cell proliferation, but induced increased interstitial cell proliferation. Previous studies have indicated that chronic heat stress can stimulate Leydig cells in interstitial proliferation (32, 33). It one study, it was shown that the number of Leydig cells in the heat-exposure group increased by 50% compared with the control group, which happened through the stimulation of cyclin proteins including proliferating cell nuclear antigen and cyclin D3. In addition, the Leydig cell's production of testosterone hormone is also interrupted because lipid metabolism is interrupted, leading to a decrease in spermatogenesis (34).

Heat stress increased interstitial fibrosis with increased expression of FSP-1, collagen I, and III in the mice exposed to high temperatures. It was previously observed that heat stress degenerated germ cells in the testicles but insignificantly affected somatic cells, decreased germ epithelial thickness, and increased testicular tissue fibrosis (35). Inflammation involves 3 processes: increasing blood supply to the damaged site, increasing capillary permeability, and increasing leukocytes into the tissue. Failure to remove the inflammatory agent will enhance the activation of macrophages, lymphocytes, and other cells that jointly produce cytokines and coordinate activities. If this process also fails to resolve the agent that will cause chronic inflammation in which inflammation and repair occur simultaneously, the result is that cytokines rise above normal levels with oxidative stress due to inflammation damaging spermatogenesis and sperm DNA, and resulting in apoptosis (29).

Immunohistochemical staining showed that the number of FSP-1-positive fibroblasts in the testicular interstitial tissue was significantly increased in the 40 C-exposed group compared to those in the control and 37 C groups. Previous research results on morphological and histological features in ram testicular heat stress showed initial testicular degeneration and mild multifocal interstitial ﬁbrosis (8). The upregulation of fibroblast-specific protein 1 has been determined to be associated with the accumulation of myofibroblasts in inflamed and fibrotic kidneys (36).

## 5. Conclusion

In conclusion, heat stress adversely affects the testicular tissue and spermatogenesis. Chronic scrotal heat stress causes inflammation and leads to testicular interstitial fibrosis.

##  Conflict of Interest

The authors declare that there is no conflict of interest.
